# Carbonate of Lead in Burns and Scalds

**Published:** 1845-12-01

**Authors:** 


*Carbonate of Lead in Burns and Scalds.*—Prof. Gross, in the Bulletin of Medical Science, and Western Journal of Medicine, recommends in strong terms, the carb. of lead, or common white paint, in burns and scalds. It should be made quite liquid with linseed or olive oil, and applied by means of a camel's hair pencil brush, or a soft linen mop. He also has found it useful as an application to irritable blisters, superficial ulcerations, excoriations, chilblains, &c. In connection with this, we would state that we have found the common red lead, made into a paste with linseed oil, an excellent application to the ulcerated legs, so common in alms-houses, and among the intemperate poor.

